# Glycosaminoglycan Interactions with Chemokines Add Complexity to a Complex System

**DOI:** 10.3390/ph10030070

**Published:** 2017-08-09

**Authors:** Amanda E. I. Proudfoot, Zoë Johnson, Pauline Bonvin, Tracy M. Handel

**Affiliations:** 1 Novimmune SA, 1228 Plan-les-Ouates, Switzerland; zjohnson@novimmune.com; 2 Novartis Pharma Schweiz A.G., Suurstoffi 14, 6343 Rotkreuz, Switzerland; pauline.bonvin@novartis.com; 3 Skaggs School of Pharmacy and Pharmaceutical Sciences, University of California, San Diego, La Jolla, CA 92093, USA

**Keywords:** chemokines, glycosaminoglycans/GAGs, heparan sulfate, chemokine therapeutics, chemokine structure, chemokine oligomerization

## Abstract

Chemokines have two types of interactions that function cooperatively to control cell migration. Chemokine receptors on migrating cells integrate signals initiated upon chemokine binding to promote cell movement. Interactions with glycosaminoglycans (GAGs) localize chemokines on and near cell surfaces and the extracellular matrix to provide direction to the cell movement. The matrix of interacting chemokine–receptor partners has been known for some time, precise signaling and trafficking properties of many chemokine–receptor pairs have been characterized, and recent structural information has revealed atomic level detail on chemokine–receptor recognition and activation. However, precise knowledge of the interactions of chemokines with GAGs has lagged far behind such that a single paradigm of GAG presentation on surfaces is generally applied to all chemokines. This review summarizes accumulating evidence which suggests that there is a great deal of diversity and specificity in these interactions, that GAG interactions help fine-tune the function of chemokines, and that GAGs have other roles in chemokine biology beyond localization and surface presentation. This suggests that chemokine–GAG interactions add complexity to the already complex functions of the receptors and ligands.

## 1. Introduction

Chemokines have been known to interact with glycosaminoglycans (GAGs) for more than 40 years, since the discovery of Platelet Factor 4 (PF-4, now referred to as CXCL4). CXCL4 was best known for its role in neutralizing heparin in the context of coagulation [1] and this interaction ultimately enabled its isolation by heparin affinity chromatography [2]. When γ-interferon inducible cytokine (IP-10/CXCL10) was cloned in 1985 [3], a common pattern of four cysteine residues was noted in CXCL10, CXCL4, and the previously identified platelet-derived protein β-thromboglobulin/CXCL7 [4], and led to the suggestion that these proteins might belong to a common family of inflammatory mediators [3]. With the cloning and functional characterization of interleukin-8 (IL-8/CXCL8) as a neutrophil chemoattractant in the late 1980s, the role of this family of proteins in directing cell migration was firmly established, and led to their classification as chemokines (derived from *chemo*attractant cyto*kines*) [5,6]. The signature cysteine motif facilitated the identification of many additional members of the chemokine family, which is now the largest cytokine sub-class, with approximately 50 members [7,8].

Although it was initially thought that soluble chemokines promoted cell migration, this notion was challenged in 1992, and an alternative hypothesis was put forward suggesting that cell migration occurs along gradients of chemokines bound to substrates such as endothelial cells or the extracellular matrix (ECM) [9,10]. Support for a haptotactic mechanism came shortly thereafter with the identification of heparan sulfate (HS) as a plausible component of endothelial cells and the ECM that could facilitate the creation of solid phase gradients [11]. CXCL8 was subsequently shown to be associated with endothelial cell (EC) projections in vivo; moreover, the presence of an intact GAG binding domain at its C-terminus was required for EC presentation and transcytosis of the chemokine, and correspondingly, the induction of neutrophil migration [12]. In more recent studies, tissue bound gradients of CXCL8 have been observed in vivo in zebrafish, with neutrophil migration dependent on the ability of CXCL8 to bind HS [13]. HS-dependent gradients of the chemokine, CCL21, have also been directly visualized within lymphatic vessels in mouse skin, and shown to be required for guiding dendritic cells toward the vessels, thereby firmly establishing the concept of haptotaxis along GAG-immobilized sources of chemokine [14]. 

**Figure 1:**
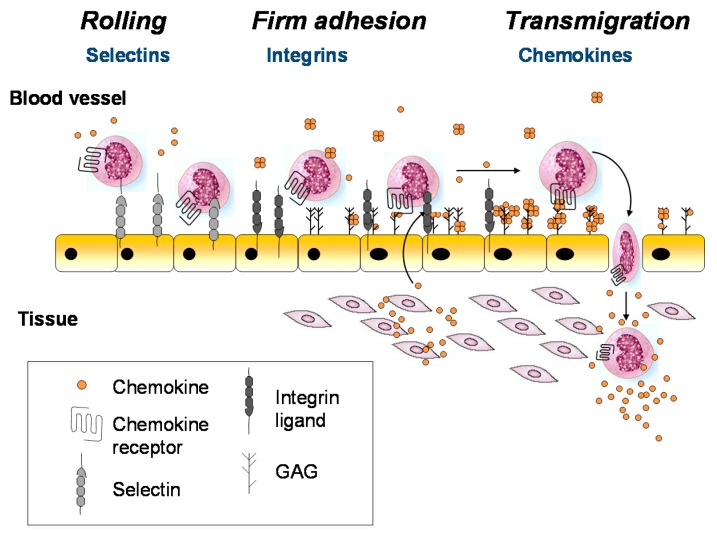
The role of GAGs in cell recruitment. Chemokines (yellow circles) produced by the underlying tissue are immobilized on cell surface GAGs. Circulating leukocytes first roll upon interaction with selectins (pale gray symbols), followed by firm adhesion to integrin ligands (dark gray symbols) following integrin activation on the leukocytes after which they transmigrate into the tissue. Although chemokines are depicted as a single species, many different chemokines may be involved.

The above and other seminal studies support the paradigm illustrated in Figure 1, where GAGs and chemokine receptors both function as chemokine-interacting partners to promote cell migration [15,16,17,18,19]. According to this mechanism, chemokines are secreted from the blood vessel wall or underlying tissue in response to inflammatory signals (e.g., infection and damage), transported to the luminal surface of the endothelial cells, and immobilized on the GAG chains of endothelial proteoglycans. Bound to GAGs, the chemokines are concentrated at the source and form an immobilized gradient that provides directional signals to guide the migration of leukocytes towards the inflammatory site. In this scenario, infiltrating leukocytes first roll along the endothelial cell surface due to weak interactions with adhesion molecules such as selectins [20,21]. Once they encounter chemokines at or near the source, the chemokines engage their cognate chemokine receptors on the surface of leukocytes, resulting in leukocyte arrest via integrin activation, followed by diapedesis through the endothelium to resolve the physiological insult [20,21,22]. This glycosaminoglycan-mediated mechanism for spatially restricting the encounter between chemokine and receptor (at a surface near the source) is thought to prevent premature activation of leukocytes before reaching the inflammatory site [10,23]. 

The type of leukocyte recruited during an inflammatory response is determined in large part by the nature of the secreted chemokines and corresponding receptors expressed on the migrating cells [7,8,24]. Thus, a large number of molecular players make up the chemokine network (discussed below) to selectively control the migration of specific leukocyte subpopulations towards specific tissues and microenvironments. However, in addition to the chemokine–receptor interaction, chemokine–GAG interactions may impose another level of control, adding complexity to an already complex system [15,16,17,18,19]. In this review we summarize the structural diversity of chemokine–GAG interactions, which suggests chemokine-specific regulation by GAGs. These diverse interactions not only regulate the formation and location of the chemokine gradient, but also its physical properties (e.g., composition, steepness and duration [14,25,26,27,28,29]). We also highlight emerging evidence for the involvement of chemokine–GAG interactions in other aspects of chemokine function, such as chemokine stability, negative and positive regulation of receptor activation, and remodeling the cellular glycocalyx, potentially to facilitate cell–cell interactions and transcytosis [30,31,32,33,34,35,36]. Finally, we address the implications of chemokine–GAG interactions on the development of therapeutics, which may explain puzzling results observed with anti-chemokine antibodies [37].

## 2. Overview of the Chemokine–Receptor and Chemokine–GAG Interactome

Approximately 50 human chemokines have been identified [7,8], most of which promote cell migration. They share a characteristic pattern of cysteine residues that defines them as belonging to CC, CXC, CX3C or C subfamilies, and led to the modern chemokine/receptor nomenclature (e.g., CXCL8, previously called IL-8, is a CXC ligand; and CCL2, previously called MCP-1, is a CC chemokine). Twenty-three human receptors have also been discovered [7,8], 18 of which belong to the G protein-coupled receptor (GPCR) family of receptors (e.g., CXCR1 belongs to the CXC receptor family), while five are “atypical receptors” (ACKR1-4, CCRL2) that neither couple to G proteins nor induce cell migration, but have other functions such as chemokine scavenging and transport [38,39]. Many chemokines bind multiple receptors and similarly many receptors bind several chemokines, leading to a dense network of interacting partners. The system is further complicated by the fact that the interactions can be regulated by post-translational modifications (PTMs, such as tyrosine sulfation), alternative splicing of the receptors, proteolysis and other PTMs of the ligands, ligand and receptor dimerization, and many other mechanisms [8]. 

Much less is known about precise chemokine–GAG interacting partners because of the many challenges of studying carbohydrates at the biochemical and cellular level. This is largely due to the heterogeneous non-template nature of GAGs [17,19,40]. GAGs are long, linear, sulfated and highly charged heterogeneous polysaccharides that are expressed throughout the body. They consist of repeating disaccharide units, and form chains that range from one to over a hundred disaccharide units, thus representing incredible biological diversity. According to an estimate by Shriver and colleagues, a GAG containing six disaccharide units could theoretically encode for more than 12 billion different sequences, which is 100 times greater than that of a hexapeptide and two million times greater than DNA [41]. As a consequence, the composition and structures of relevant chemokine–GAG complexes are likely quite heterogeneous and large in number, even for a single chemokine. 

GAGs are also difficult to produce synthetically and to characterize analytically [42,43,44]; thus even though heparan sulfate (HS) is biologically more relevant, heparin is often used for in vitro studies due to the availability of reagents including short GAGs that are size-defined and homogeneous in composition [45,46]. Demonstrating that specific chemokine–GAG complexes are relevant in vivo is even more challenging because antibodies to specific GAG sequences are not available and siRNA strategies against specific enzymes broadly affect GAG composition. Nevertheless two decades of mutagenesis, biophysical, structural and in vivo studies, have lead to a basic understanding of the structural principles of chemokine–GAG interactions, their functional consequences, and strategies to exploit or interfere with their interactions for therapeutic purposes [16,17,47]. 

## 3. Structural Biology of Chemokine–Glycosaminoglycan Interactions

### 3.1. Chemokine Tertiary Structure, Receptor-Binding Domains and Glycosaminoglycan-Binding Epitopes

**Figure 2:**
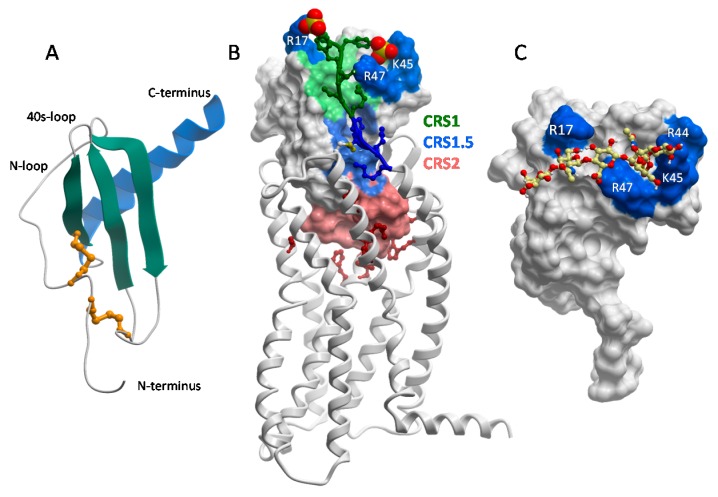
Chemokine tertiary structure, chemokine–receptor interactions, and chemokine–glycosaminoglycan interactions. (**A**) Structure of a typical chemokine, illustrated by a monomeric subunit of CXCL8 (PDB ID 1IL8 [48]). (**B**) Structure of CCR5 bound to a variant of CCL5 with a modified N-terminus (PDB ID 5UIW [52]). Chemokine recognition sites 1, 1.5 and 2 (CRS1, CRS1.5 and CRS2) are highlighted in green, blue and salmon. In CRS1, residues that contribute to GAG binding are highlighted in dark blue (R17 and 40s-loop BBXB motif residues R44 (not visible), K45 and R47). Tyrosine sulfates are highlighted as orange and yellow spheres interacting with the 40s-loop cluster and R17. (**C**) Model of CCL5 in complex with a chondroitin sulfate (CS) hexasaccharide from paramagnetic relaxation enhancement and intra- and intermolecular nuclear Overhauser effect constraints (supplementary data to [56]). GAG binding residues R17, R44, K45 and R47 are highlighted in dark blue. Comparison with (**B**) shows that GAG and receptor utilize similar epitopes on the chemokine core domain (CRS1) and sulfation is involved in both cases. Consequently, binding of GAGs and receptors to chemokines are generally mutually exclusive. Panels (**A**–**C**) are not to scale.

The structure of CXCL8 [48] revealed what has proven to be the consensus fold of all chemokines known to date [7,8,49]. It consists of a disordered N-terminus, an irregular loop referred to as the “N-loop”, a three stranded β-sheet connected by loops (the 30s-, 40s- and 50s-loop), and a C-terminal helix. The “core” globular domain of the chemokine (everything beyond the N-terminus) is stapled to the N-terminus via one, and usually two, disulfide bonds (Figure 2A). The N-terminus of the chemokine functions as a key signaling domain, and binds within the pocket of chemokine receptors where it interacts with receptor transmembrane helices (chemokine recognition site 2, CRS2) to promote activation, similar to small molecule activation of GPCRs [50,51,52]. The chemokine core domain interacts with the receptor N-termini and extracellular loops (CRS1 and CRS1.5) and functions primarily as a binding determinant (Figure 2B). However, it also serves as the binding site for GAGs [53,54,55,56] (Figure 2C).

With the exception of CCL3/MIP-1α and CCL4/MIP-1β, which are acidic, chemokines are highly basic proteins. As a consequence, they tend to have the highest affinity for HS and heparin over GAGs with lower sulfate content (e.g., chondroitin sulfate (CS), dermatan sulfate (DS) [35]). The GAG-binding epitopes for many chemokines have been identified and unsurprisingly are dominated by basic residues [17,49,53,55,57,58,59,60,61,62,63]. In several chemokines, GAG binding motifs are defined by characteristic “BBXB” sequence motifs (where B is a basic and X is any amino acid). Taking the example of CCL5, the 40s-loop BBXB cluster, ^44^RKNR^47^, was originally shown to be the most important GAG binding epitope by in vitro and in vivo experiments [47,58] (Figure 2C). In the case of CCL2, the main contributors are non-BBXB residues R18, K19, R24 and K49, which form a basic patch on the chemokine core domain [55]. In both CCL2 and CCL5, these basic residues are also important for receptor binding, and interestingly, involve interactions with sulfate groups on both the GAG and the receptors (which are modified by tyrosine sulfation [64,65]). In general there is a great deal of overlap between GAG binding residues and receptor binding residues on the chemokine core domain, which suggests that a single chemokine subunit cannot simultaneously interact with receptors and GAGs [54,55,56,61] (Figure 2B,C). 

### 3.2. Chemokines Bind Receptors as Monomers but Many Chemokines Bind GAGs as Oligomers

**Figure 3:**
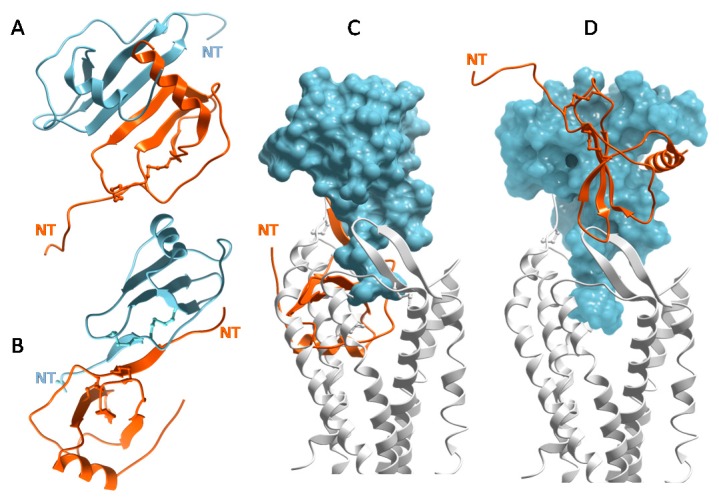
Chemokine oligomers and their ability to bind chemokine receptors, or not. (**A**) Structure of a typical CXC dimer, illustrated by CXCL12 (PDB ID 3GV3) with individual subunits colored in blue and orange. (**B**) Structure of a typical CC dimer, illustrated by vMIP-II (PDB ID 2FHT) with individual subunits colored in blue and orange. (**C**) Docked model of the vMIP-II dimer to CXCR4 shows that CC dimers are sterically incompatible to bind receptor; the orange subunit completely overlaps and clashes with the receptor. (**D**) Docked model of the CXCL12 dimer with CXCR4 shows that CXC dimers are compatible with receptor binding. Models from (**C**,**D**) are reported in [50].

Numerous chemokine structures have now been solved and show the tendency of many chemokines to oligomerize [16,49,66]. CXCL8 and other CXC chemokines form a characteristic “CXC dimer” driven by the antiparallel association of β1 strands from two separate subunits of chemokine monomers (Figure 3A) [48]. CC chemokines such as CCL2/MCP-1 adopt the canonical tertiary structure (Figure 2A), but have a different quaternary fold (Figure 3B) [67,68]. They form elongated “CC dimers”, stabilized by the formation of an antiparallel β-sheet between residues proximal to the first two Cys residues of the component dimer subunits. At first, chemokine dimers were thought to contribute to the selectivity of CXC chemokines for CXC receptors and CC chemokines for CC receptors [67]. However, it was subsequently shown with oligomerization-impaired mutants that chemokines bind and activate receptors as monomers, which was confirmed by cell based studies [69,70,71] and by recent X-ray structures of intact chemokine–receptor complexes [50,51,52] (Figure 2B). Moreover, when the first structure of a chemokine–receptor complex was determined, it was clear that CC dimers cannot bind receptors due to steric incompatibility [50] (Figure 3C), which is consistent with experimental studies [72,73]. Disulfide cross-linked CXC chemokine dimers are compatible with receptor binding (Figure 3D) and have been shown to be functional in vitro and in vivo; however, these engineered dimers typically show lower affinity and partial agonism compared to their WT counterparts in vitro [74,75,76,77,78]. In summary, chemokine monomers function as full agonists of receptors, CC chemokine dimers cannot bind receptors and while CXC dimers are sterically compatible with receptor binding, the role of such complexes in vivo is not known.

By contrast, chemokine oligomers are important for the interactions of many (but not all) chemokines with GAGs. In vitro biochemical and biophysical studies showed that chemokines oligomerize on GAGs and that GAGs stabilize dimers and higher order chemokine oligomers [45,55,79,80]. This suggests that binding of chemokines to GAGs and chemokine oligomerization are mutually re-enforcing processes, which would provide a mechanism for concentrating chemokines near inflammatory sites. Importantly, in vivo studies of chemokine-induced cell migration also demonstrated the importance of chemokine oligomerization and GAG binding [81]; in these studies GAG-binding deficient mutants and monomeric mutants of CC chemokines failed to stimulate cell migration into the peritoneal cavity of mice. In similar in vivo peritoneal recruitment assays, obligate CXC chemokine monomers were active; nevertheless, since they were less active than their WT counterparts, the data also support the importance of chemokine oligomerization [74,75]. Taken together, the studies collectively suggest that for many chemokines, both oligomerization and GAG binding are critical for regulating cell migration in vivo [27,74,75,81,82]. 

### 3.3. Chemokines Have a Wide Range of Oligomerization Propensities and Affinities for GAGs

**Figure 4:**
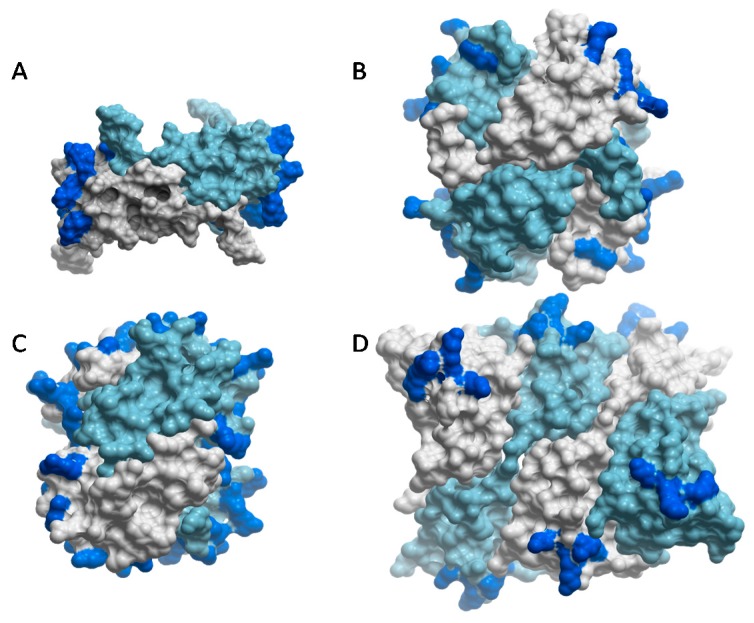
Chemokines adopt a wide range of oligomeric structures, sometimes in response to different GAGs. Structures are shown with the different chemokine subunits color-coded in sky blue and gray, and the main GAG binding residues highlighted in dark blue. (**A**) The CCL2 dimer, which binds to HS (PDB ID 1DOM [68]); residues highlighted include R18, K19, R24, K49. K58, H66 [55]. (**B**) The CCL2 tetramer, which binds to heparin (PDB ID 1DOL [86]); residues highlighted include R18, K19, R24, K49. K58, H66 [55]. (**C**) The CXCL4 tetramer, which binds to heparin (PDB ID 1RHP [85]); residues highlighted include R20, R22, K46, R49, K61, K62, K65, K66 [87]. (**D**) The CCL5 polymer (PDB ID 5DNF [84]); residues highlighted include K55, K56 and R59 [58,88].

Chemokine oligomerization is not restricted to dimers; instead they adopt a spectrum of oligomerization states from monomers (e.g., CCL7/MCP-3) to tetramers and polymers (e.g., CXCL4 and CCL5, respectively) [45,83,84,85] (Figure 4). For those that do self-assemble, the ability to oligomerize has been show to be important for their GAG-binding affinities. This has been demonstrated with oligomerization-deficient variants, which have markedly reduced affinities for heparin and HS in vitro, and corresponding reduced abilities to accumulate on cell surfaces compared to WT chemokines [31,45,81]. By contrast, although CCL7/MCP-3 does not oligomerize, it still binds GAGs because it has a high density of GAG binding epitopes within its tertiary structure compared to the highly homologous but oligomerizing chemokine, CCL2 [83].

Chemokines also have a wide range of affinities for GAGs. CXCL4, CXCL11, CCL5 and CCL21 have very high affinities for GAGs whereas CCL2 and CXCL8 have intermediate affinities, and the affinities of CCL3 and CCL4, the two uniquely acidic chemokines, are weak [45,89]. This is consistent with studies which suggest that only a subset of chemokines bind efficiently to cell surface and ECM GAGs [89]. Together, the GAG-binding affinities of chemokines coupled with their different oligomerization propensities affect the amount of chemokine that accumulates on GAGs, as well as the persistence of the chemokines on GAGs [31,45]. The expectation is that these in vitro results should translate into differences in the nature, shape and duration of chemokine–glycosaminoglycan gradients in vivo [28,29]. 

### 3.4. Some Chemokines Bind GAGs through C-Terminal Tail Interactions

Whereas CXCL12α, the most well-characterized of the CXCL12 isoforms, forms dimers and polymers [90,91], the CXCL12 splice variant, CXCL12γ, is not known to oligomerize. Instead, CXCL12γ disordered, approximately 30 amino acid C-terminal extension with a tight array of BBXB motifs that confers even higher affinity for GAGs than CXCL12α [62,92]. Similarly, the CCR7 ligand, CCL21, has an extended, basic C-terminal region of approximately 40 residues, which is required for its immobilization on GAGs and the recruitment of dendritic cells [14]. Along with diverse oligomers formed by chemokines, these alternative GAG-interaction mechanisms imply chemokine-specific and GAG-specific control of cell migration [92]. 

### 3.5. Specificity and Structural Plasticity in Chemokine–GAG Interactions

An important but challenging problem is identifying the structure of GAGs that are recognized by specific chemokines. Given the diversity of GAG sequences, it is likely that chemokines recognize numerous GAGs. As reviewed by Monneau and coworkers [19], little is known about this topic, because GAGs are not as easily isolated and analytically characterized, or amenable to structure-function analysis as routinely performed for proteins. However, some generalizations are worth noting. First, longer GAGs (e.g., roughly 18–20 monosaccharides for CCL3 and CXCL12) tend to have higher affinity for chemokines than short GAGs. This is expected since longer GAGs would be able to bridge multiple GAG-binding epitopes including those between different chemokine subunits on oligomers [35,36,93] (Figure 4). As described above, GAG sulfation is also important. For example, 2-O-desulfation of heparin causes a significant loss of affinity for many chemokines. However, the distribution of sulfates appears to be more important than the actual average sulfate content. In a study by Dyer and coworkers [45], a panel of chemokines was shown to have similar affinities for heparin (characterized as having an average of 2.3 sulfate groups per disaccharide, where >90% of the disaccharides have at least one sulfate) and HS (characterized as having an average of 0.8 sulfates per disaccharide, where 55% of the disaccharides were unsulfated). By contrast, chondroitin sulfate-A (CS-A), which has a relatively low content and uniform distribution of sulfates (characterized as having ~0.7 sulfates per disaccharide, similar to HS, where ~84% of the disaccharides are sulfated, similar to heparin), had a significantly reduced affinity for chemokines compared to HS and heparin. In fact, only the subset of chemokines with the highest affinity for HS and heparin were able to bind CS-A [45]. Even the precise position of a single sulfate can be important, as demonstrated in a study where the presence or absence of a 6-O-sulfate interchanged the ability of a dodecasaccharide to inhibit the biological function of CXCL8 vs. CXCL12 [94]. These and other data suggest that there is significant specificity in chemokine–GAG recognition [19,94,95]. 

Despite evidence for specificity, it is becoming clear from structural data that chemokine–GAG interactions are not defined by single structures; instead, these complexes are characterized by structural plasticity which may enable a single chemokine to recognize multiple GAG sequences. For example, different oligomeric structures of the same chemokine can present different spatial geometries of its GAG binding residues, enabling recognition of different GAGs. Along these lines, HS prefers CCL2 dimers, whereas heparin binds and stabilizes CCL2 tetramers [55,83]. These preferences can be rationalized in terms of the distribution of GAG-binding epitopes on the chemokine oligomers, and the corresponding pattern of sulfation on the GAGs [55,57,93]. In the CCL2 dimer, the basic GAG binding clusters are separated by ~45 Å, while HS typically consists of highly sulfated regions connected by intervening N-acetylated regions (e.g., SAS domains) [19,40]. By associating with the CCL2 dimer, the spacing of the sulfated domains on HS presumably overlap with the basic epitopes of CCL2, thereby stabilizing the complex [55] (Figure 4A), similar to complexes of CCL3 with HS (90). By contrast, in the tetrameric arrangement of CCL2, the GAG-binding residues form an almost continuous basic epitope that wraps around the chemokine surface and would better complement the more uniformly sulfated nature of heparin than the epitope distribution in the chemokine dimer [55] (Figure 4B). A similar continuous distribution of epitopes on the CXCL4 tetramer has also been suggested to facilitate its interaction with heparin [87] (Figure 4C). Other chemokines such as CXCL10 and CXCL12, have been captured in different oligomerization states by crystallography [90,96,97], which may reflect an ability of these chemokines to bind different GAGs by adopting different oligomer forms. 

Structural plasticity, whereby different epitopes are involved in GAG binding, depending on the oligomerization state of the chemokine, is also apparent from studies of CCL5 [56,58,98,99]. Three structures of a CCL5 dimer with sulfated disaccharides showed contacts between the disaccharides and the 40s-loop BBXB cluster, as well unexpected interactions with the 30s-loop and N-terminus of the chemokine [58,98]. The 40s-loop and N-terminal interactions of the dimer were also observed in the structure of the CCL5 dimer with a CS hexasaccharide [56] (Figure 3C). However, in the structure of a polymeric CCL5 complex containing a heparin hexasaccharide, the 50s-loop basic motif, ^55^KKWVR^59^, was shown to be the main binding site for heparin while the 40s-loop motif was largely buried [84] (Figure 4D). These data suggest oligomerization-dependent specificity in chemokine–GAG interactions, with the 40s-loop and 50s-loop playing roles in different contexts. In fact, one study attributes different functions to the 40s-loop and 50s-loop GAG interactions, and emphasizes the importance of chemokine presentation in mediating these different functional roles [88]. 

Certain chemokines that utilize unstructured C-terminal tails for GAG binding (e.g., CCL21, CXCL12γ) may be particularly promiscuous in their ability to bind numerous GAGs because of the likelihood that the tails adopt multiple geometries. Chemokines with high-density GAG binding domains (e.g., CCL7) may also have a high degree of structural plasticity even if they do not oligomerize [83]. Structural plasticity has the potential to expand the range of chemokine interactions beyond what might be expected for these small proteins, and enable chemokines to adapt to local GAG environments, potentially with differentiating functional consequences, as suggested by the CCL5 study [88].

### 3.6. Heterodimerization and Post-Translational Modifications of Chemokines–Mechanisms for Regulating Chemokine–GAG Interactions

In addition to forming homo-oligomers, chemokines have been shown to heterodimerize [100,101,102,103,104,105]. Since multiple chemokines are frequently expressed at sites of inflammation, heterodimerization would offer a potential mechanism for regulating GAG binding, and indirectly, receptor activation. Using NMR, Nesmelova and coworkers demonstrated that incubating CXCL4 tetramers and CXCL8 dimers together resulted in subunit exchange and the formation of CXCL4:CXCL8 heterodimers [104]. Similarly, incubation of CCL2 with CCL8 resulted in a strong preference for CCL2:CCL8 heterodimers over the corresponding homodimers [100]. CC:CXC heterocomplexes involving CCL5 and CXCL4 have also been observed and are thought to have functional consequences in amplifying leukocyte arrest responses to CCL5 [102]. As shown by Crown and coworkers, GAGs can also stabilize the formation of chemokine heterocomplexes [100], and in principle heterocomplexes can modulate GAG-binding affinity [100,106] and possibly even specificity. For example, the isolation of GAG-bound heterocomplexes containing CCL3, CCL4 and CCL5 with sulfated proteoglycans [106] may represent an example where the high affinity of CCL5 dominates the weak affinities of CCL3 and CCL4 such that CCL3 and CCL4 assemble with CCL5 in a GAG complex that might not otherwise form for CCL3 or CCL4 alone. 

Post-translational modifications (PTMs) of chemokines also affect GAG interactions. Citrullination of Arg5 in CXCL8 by peptidylarginine deiminase reduces its affinity for GAGs [107] as does nitration of Tyr13, Tyr28 and Trp59 in CCL2 [108]. Since Tyr13 is important for CCL2 dimerization [70,86] while Tyr28 is involved in the tetramer interface [86], nitration of these residues almost certainly disrupts GAG binding by destabilizing CCL2 oligomers. Thus, modulating the interaction of chemokine–GAG interactions via PTMs adds yet another mechanism for regulating cell migration in a chemokine-dependent manner.

## 4. Are Chemokine Receptors Activated by GAG-Immobilized Chemokine, Soluble Chemokine, or Both?

Despite the emphasis on haptotactic gradients, a long-standing issue has been the role of immobilized versus soluble gradients of chemokines, as well as if, and how, GAG-bound chemokines activate receptors. Specifically: (i) Are receptors on leukocytes activated by chemokines simultaneously bound to GAGs on endothelial cells or the ECM (i.e., immobilized haptotactic gradients)? (ii) Are chemokines concentrated in immobilized depots by GAGs but released in soluble form to activate receptors and cause cell migration close to the source (a hybrid haptotactic/chemotactic gradient)? (iii) Do some chemokines have little or no interaction with GAGs and simply create a gradient by diffusion from the source (e.g., chemotaxis)? This issue awaits a definitive answer, but current data suggest that all three scenarios may be relevant depending on chemokine, and that different types of gradients may function together to control cell migration in vivo [28,29]. It is clear that cell migration to soluble chemokines is feasible since simple Boyden chambers that rely on soluble gradients are routinely used by most laboratories to monitor cell migration. Migration to essentially immobilized chemokine has also been observed in some [28] but not other [109] experiments where differences may reflect the completely different experimental setups and/or chemokine systems under study. The development of sophisticated microfluidic devices and biomimetic surfaces that enable precise control over the composition, shape and stability of chemokine gradients [28,109,110], will undoubtedly lead to a deeper understanding of this complex issue and complement in vivo studies where achieving such precise control would be much more challenging. 

In the meantime, one can hypothesize based on structural and affinity information, what types of gradients might be feasible for different chemokines. Simultaneous binding of chemokine to receptor and GAG should be possible for CCL21 and CXCL12γ since the extended C-terminal tails of these chemokines is expected to orient away from the receptor in the receptor–chemokine complex, making it accessible to GAG. This hypothesis is supported by the fact that a CCL21 variant, immobilized through a PEG-biotin tag to a streptavidin-biotin surface and patterned in a gradient, promotes dendritic cell (DC) adhesion and migration [28]. Whether this happens in vivo, or the chemokine is released from GAGs prior to receptor engagement, remains to be seen. However, in vitro results clearly support the idea that soluble and haptotactic gradients of CCL19 and CCL21, respectively, work together to recruit CCR7 expressing DCs [14,25,28,111].

For other chemokines lacking a tail, simultaneous interactions of chemokine with receptor and GAG seems less likely. As described above, the extensive overlap between GAG binding sites and receptor binding sites on the surface of chemokine monomers, suggests that the interactions are mutually exclusive (Figure 2B,C). Arguments could be made that the N-terminus of the chemokine can bind in the receptor binding pocket (CRS2) while the chemokine core domain (CRS1) binds to glycosaminoglycans prior to engaging the receptor N-terminus. However, given the low affinity of monomeric forms of oligomerizing chemokines for GAGs [45], this scenario seems highly unlikely. It has also been suggested that oligomerized chemokines might be able to simultaneously interact with receptors and GAGs using different subunits of the oligomers [55]; however, this is not possible for CC chemokines because CC dimers cannot physically bind receptors [50,72,73] (Figure 3C). While CXC chemokine dimers are compatible with receptor binding (Figure 3D) [50,74,75,76,77,80], again, the affinity of a single subunit of a chemokine dimer for GAG is low [45]; thus simultaneous interaction seems unlikely. We are also unaware of higher order CXC chemokine oligomers that would be physically compatible with receptor binding. Majumdar and colleagues suggested that while chemokines may remain bound to GAGs while being sensed by receptor, that it is also possible that GAGs attract dense “clouds” of chemokines, which are then sensed in their soluble state [29]. Structural data are most consistent with this explanation for the majority of chemokines; in other words, GAG interactions provide a mechanism for concentrating and localizing chemokines, but chemokines most likely dissociate before interacting with receptor. This contradicts a previous hypothesis proposed by the authors suggesting that the bound form might be the active form [55,81]. However, it is consistent with studies of a neutralizing anti-CXCL10 antibody (described below) that supports the concept of a cloud of chemokine released from a GAG-bound cluster [37].

As suggested in early studies [89], some chemokines may only form soluble gradients which is consistent with their low affinities measured in vitro [45]. Recent studies also suggest that endothelial presentation is not necessary for some chemokines and cells. As demonstrated by Stoler-Barak and colleagues, activated T cells can obtain transmigration cues directly from intra-endothelial chemokine stores [112]. 

## 5. Beyond Chemokine Gradients: Functional Effects of Chemokine–GAG Interactions

Most literature reports discuss the role of chemokine–GAG interactions in the context of gradient formation. However, GAG interactions are involved in many other aspects of chemokine biology, as reviewed in this section.

### 5.1. Positive and Negative Regulation of Receptor-Mediated Functional Responses

Several reports have documented the ability of soluble, exogenously added GAGs, to inhibit the function of many chemokines in assays of receptor binding and activation [35,94]. Now that structures are available, the reason is clear—the GAG binding epitopes and receptor binding epitopes of chemokines overlap heavily [54,55,56,61], as illustrated in Figure 2B,C. This means that while GAGs and receptors work together to promote cell migration, they can also have opposing functions. Cells shed GAGs during many pathological situations [40], which, in principle, could inhibit chemokine–receptor interactions and consequently inflammatory responses. On the other hand, soluble HS has been reported to act as a coreceptor for CCL21 [113] and to enhance neutrophil responses to CXCL8 [11]. Inhibition of chemokine function is the more commonly documented result, however, and this has inspired efforts to develop small GAG mimetics as receptor antagonists [114,115], which is discussed in Section 6.

Several studies have reported that combinations of chemokines, including chemokines from different subfamilies, and those that activate different receptors, can potentiate the functional responses of individual chemokines. For example, chemokines CCL2, CCL3 and CCL8 (all monocyte chemoattractant proteins) were shown to function cooperatively with CXCL8 in an assay of neutrophil chemotaxis, when none of the CC chemokines showed any function on their own. CXCL8 also showed synergy with CXCL4 and CXCL12, in neutrophil migration [116]. Similarly, CCL19 and CCL21, which do not have receptors on monocytes, caused migration and cellular responses of CCR2 agonists at much lower concentrations than the CCR2 agonists alone [117]. These and many additional examples of synergistic chemokine activities [102,118,119,120,121] led to the hypothesis that in inflammatory situations, an abundance of different chemokines could produce a reduction in the threshold for cell migration and activation [122], thereby invoking more robust cell responses at lower chemokine concentrations than would be anticipated from single chemokine–receptor pairs. 

In some reports, this functional synergy has been attributed to positive crosstalk between intracellular signaling pathways downstream of receptors [116,119] or by formation of hetero-complexes between a receptor, its cognate chemokine, and a non-cognate chemokine [102,122]. However, other reports suggest that interactions with GAGs can produce synergistic effects of chemokines on receptor activation [32]. In the latter scenario, chemokines that do not activate a given receptor, potentiate the activity of a bona fide receptor agonist by displacing the agonist from GAGs, and increasing its effective concentration for binding receptor. In support of this competitive binding mechanism, functional cooperativity was demonstrated for many chemokine combinations, whereas GAG-binding deficient chemokines (still able to activate receptor) were incapable of reproducing the same synergy. 

Although not a subject of the above study, CXCL4 might be expected to show synergy with other chemokines due to its exceptionally high affinity for GAGs, and corresponding ability to competitively displace many chemokines. Since it is a weak chemoattractant [123] but a strong GAG-binder [45], having a significant role in regulating chemokine function by modulating chemokine–GAG interactions is intuitively appealing. On the other hand, competitive displacement of certain chemokines from GAGs could also lead to functional inhibition by disrupting chemokine gradients, and CXCL4 could certainly also negatively regulate more weakly binding chemokines in this manner. In this regard, a negative regulatory role was postulated for CCL18, another chemokine with weak chemoattractant properties [124]. CCL18 is found at high levels in the circulation, and is further upregulated in many diseases [125,126]. Along with its ability to inhibit the function of several chemokine/receptor pairs through competitive receptor interactions, it was found to displace chemokines from heparin in vitro, which could also translate into gradient disruption in vivo [124]. Finally, since many cytokines and growth factors also rely on GAG binding, it is possible that chemokines could regulate other cytokines as well, and visa versa, through competitive displacement from GAGs.

### 5.2. GAGs Protect Chemokines from Proteolysis

Proteolysis of chemokines is a well documented mechanism for regulating the inflammatory response [127], which can be rich in proteases in addition to inflammatory stimuli. The role of chemokine N-termini as the crucial mediators of signaling makes them natural targets for proteolytic processing, where even single amino acid changes can alter the pharmacological activity of chemokines [127]. For example, limited proteolytic processing of the N-termini of CXCL8 and CCL3L1 results in enhanced chemokine activity, while it results in reduced activity of CCL7 and CCL11 [128,129,130,131] and inactivation of CXCL12 [132]. Chemokines are also proteolytically cleaved internally and at their C-termini, which regulates their activity. For example, the extended GAG-binding domain of CCL21 is cleaved by a dendritic cell-associated protease, and this modification produces a soluble version of CCL21 that can no longer induce leukocyte arrest [14,25]. GAGs can modulate these regulatory processes by protecting chemokines from proteolytic degradation [33,36,133]. The protection may be due to steric blockade of the chemokine from the protease active site in some cases. However, stabilization of chemokine oligomers may also play a role [36,84]. In the context of the CCL5 polymer, for example, the chemokine N-terminus is buried and unavailable for modification. Thus, in principle, chemokine protection from proteolysis through interactions with GAGs could regulate the intensity and duration of an inflammatory response.

### 5.3. Chemokines Modulate the Mechanical Properties of Heparan Sulfate and May Contribute to Glycocalyx Remodeling

**Figure 5:**
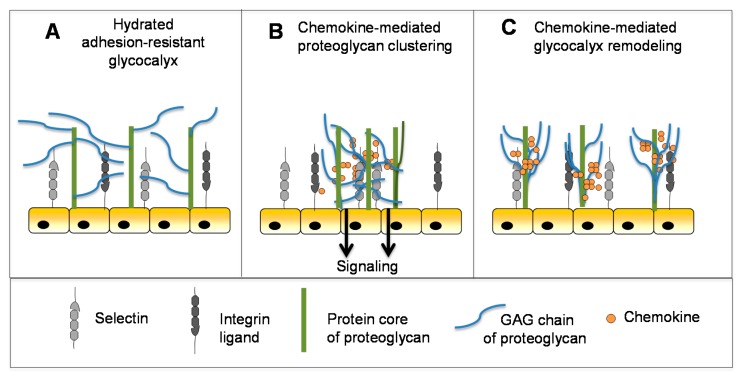
Functional effects of chemokine–GAG interactions. (**A**) The GAG chains of proteoglycans form a hydrated adhesion-resistant surface on endothelial cells that inhibits leukocyte transmigration. (**B**,**C**) Binding of chemokines to the HS chains of proteoglycans may promote: clustering of the syndecans and chemokine–receptor independent signaling (**B**); or remodeling of the endothelial glycocalyx to enable leukocyte transmigration (**C**). Figure adapted from [31].

In addition to providing a substrate for the localized accumulation and slow release of chemokines to guide leukocytes to inflammatory sites, the GAG chains of endothelial cell surface proteoglycans contribute to the physical adhesion-resistant barrier function of the glycocalyx [134,135,136]. By virtue of its physicochemical properties and thickness, the glycocalyx inhibits contact between key molecules (e.g., selectins, integrins and their ligands) that mediate cell–cell interactions required for leukocyte adhesion and transmigration [137] (Figure 5A). The molecular mechanisms that control this barrier are poorly understood but the glycocalyx is known to undergo remodeling in the context of disease and inflammation to permit close approach of endothelial cells and leukocytes [136,138,139]. Inflammatory mediators like TNF play a role in reducing the barrier thickness to promote cell adhesion and subsequent transmigration [140]. This suggests that chemokines may also play a role [31], although, at a nascent stage of understanding, novel biophysical techniques that report on the fluidity and diffusion of HS chains have shown that chemokines differentially crosslink and rigidify HS in biomimetic surrogates of cell surface proteoglycan layers [30,31]. Notably, high affinity chemokines showed the greatest propensity to crosslink and remain associated with HS chains, which was also significantly enhanced by their ability to oligomerize. Although the implications of these findings are speculative at this point, it has been suggested that HS clustering may be involved in receptor-independent signaling of chemokine through proteoglycans (Figure 5B), as has been observed for CXCL12 and CCL5 [31,34,141]. HS remodeling may also prepare the endothelial surface for localized leukocyte-endothelial adhesion events prior to leukocyte transmigration (Figure 5C) [31]. Consistent with this concept, CXCL12 and CCL5 have been shown to stimulate proteoglycan (syndecan) clustering and shedding, an important mechanism for reducing the glycocalyx barrier thickness and density to permit cell–cell contact [142,143]. This clustering may be a consequence of the ability of these chemokines to crosslink proteoglycan HS chains as demonstrated by the above in vitro experiments [30,31]. If relevant, HS/glycocalyx remodeling would likely occur concomitantly with chemokine gradient formation, and represent yet another critical role for chemokine–GAG interactions in cell migration. 

## 6. Exploiting Chemokine–GAG Interactions for Therapeutic Applications

Targeting the chemokine system for therapeutic applications has almost exclusively focused on inhibiting chemokine–receptor interactions, either with small molecules interacting with receptors, or with antibodies neutralizing the receptors or the chemokines themselves. However, in view of the essential nature of chemokine–GAG interactions for cell migration and activation, alternative strategies targeting these interactions can be envisioned to disrupt the function of the chemokine system, as described below.

### 6.1. Protein-Based Strategies

In one approach, non-signaling chemokine variants were created by mutating residues that activate receptor, concomitant with mutations that increase the strength of the GAG interaction [144,145]. Such non-signaling super-GAG-binding variants have been tested in several disease models and found to reduce inflammation in preclinical models (for example a CXCL8 variant reduced inflammation in an antigen-induced arthritis model [146]). This approach, trademarked as the CellJammer^®^ technology, is in clinical development [147].

An alternative approach was discovered by serendipity in the process of defining the GAG-binding pharmacophores of CCL5 and several other chemokines. Mutation of the BBXB motif (^44^RKNR^47^ to^44^AANA^47^) produced a CCL5 mutant incapable of recruiting cells in vivo, thereby highlighting the essential role that this interaction plays in directing cell migration [58,81]. Other chemokines with abrogated GAG binding such as CCL2, CCL7 and CXCL11 were also unable to recruit cells in vivo [55,148,149]. While these observations were important for elucidating the biological relevance of chemokine–GAG interactions, the most surprising result was that these variants had anti-inflammatory properties. Essentially, pre-treatment with these mutated proteins blocked the ability of the wild type chemokines to induce cell recruitment in vivo, which translated into reduced inflammatory symptoms in several pre-clinical disease models. For example, the ^44^AANA^47^-CCL5 variant reduced inflammatory and clinical symptoms in experimental autoimmune encephalitis (EAE), as well as in models of liver injury and fibrosis, myocardial reperfusion and atherosclerosis [47,150,151,152], and a CCL7 GAG binding mutant prolonged skin allografts [149]. However, these variants were partial or full agonists that retained some ability to activate their receptors, which in the case of CCL5, ultimately halted a discovery program prior to Phase I clinical studies.

Subsequent studies of the mechanistic basis for the inhibitory properties of ^44^AANA^47^-CCL5 re-enforced the interrelationship between GAG binding and oligomerization of chemokines, suggesting that oligomerization-deficient mutants might also be functional antagonists. Specifically, the BBXB mutation not only disrupted the ability of CCL5 to bind GAGs, but limited it to the formation of dimers rather than the polymers. Furthermore, ^44^AANA^47^-CCL5 was shown to act as a “dominant-negative” inhibitor of WT CCL5 by forming non-functional (i.e., GAG-binding deficient) heterodimers [47]. Following this observation, oligomerization-impaired mutants of CCL2, CCL4 and CCL5 were then tested and shown to be inactive in vivo [81]. As was the case for the GAG mutants, inhibition of WT proteins by the oligomerization-deficient mutant of CCL2, P8A-CCL2, was also observed. The P8A-CCL2 variant demonstrated anti-inflammatory properties in a murine model of EAE [153] as well as in a rat model of arthritis [154]. 

The inability of GAG-binding deficient and oligomerization deficient chemokines to recruit cells in vivo, does not hold for all chemokines. When administered into the lungs of mice, CXCL8 GAG mutants recruited more neutrophils than WT CXCL8 [82]. The result was attributed to the appearance of the mutants in plasma at significantly higher concentrations than WT CXCL8, due to more rapid diffusion across the extracellular matrix. Disulfide-crosslinked obligate monomers of CXCL1 and CXCL8 were also shown to be active in vivo, although less so than their WT counterparts [74,75]. Furthermore, different results have been observed when the same chemokine variant was administered in different tissue compartments (i.e., recruitment in the lung versus the peritoneal cavity [74]. Thus, a major take home message is that the GAG interactions and oligomeric properties of chemokines are finely tuned to regulate cell migration in a chemokine-specific and tissue-specific manner [27,74,75,82].

In addition to the potential of chemokines with modified GAG-binding properties as therapeutics, chemokine–GAG interactions can be exploited for stabilization and delivery purposes. An N-terminally modified variant of CCL5 was developed as a potent CCR5 antagonist and inhibitor of HIV infection [155,156]. Encapsulation of the chemokine in GAG-containing hydrogels was shown to significantly preserve its activity and facilitate its controlled released over the course of a month [157]. Other therapeutic applications of GAG-based hydrogels have been reported such as their use in sequestering chemokines to promote wound healing [158].

### 6.2. Small Molecule-Based Strategies

In view of the facts that chemokine–GAG interactions often involve binding epitopes defined by small clusters of basic residues on the chemokine, and that soluble GAGs inhibit receptors, it was hypothesized that small molecule inhibitors targeting these clusters, could be identified. Earlier proof of concept studies showed that small heparin derived saccharides could indeed block CCL5-mediated cell migration in vivo [114]. However, a tetrasaccharide was the minimum-sized fragment to show an effect, and a 15-fold excess of GAG over chemokine was needed to observe statistically significant inhibition; in other words it was not very effective. Subsequent studies explored the use of small persulfated oligosacharrides, which also showed anti-inflammatory results in some disease models [114]. However, for reasons that are unclear, these molecules induced pro-inflammatory responses in a CCL5-mediated peritoneal recruitment model. 

In contrast to small molecules directed against CCL5, promising results were obtained from a study targeting CCL20. A GAG microarray, designed to explore the specificity of various chemokines for different GAGs revealed that a synthetic monosaccharide, 2,4-*O*-di-sulfated iduronic acid (Di-S-IdoA), had high affinity for CCL20 but not for seven other chemokines [115]. When tested in a mouse model of allergic asthma, the monosaccharide caused attenuated leukocyte recruitment into the lungs and bronchoalveolar lavage fluid. Moreover, the reduced leukocyte accumulation was associated with a lack of CCL20 on the vascular endothelial cells in the treated mice, whereas robust CCL20 expression was observed in the untreated mice. What is most striking about the results is that Di-S-IdoA is only a monosaccharide, which bodes well for the potential of blocking chemokine-mediated disease with small molecule carbohydrate mimics. It may be that some chemokines and some disease contexts are easier to block than others. For example, CCL20 interactions with GAGs may be more amenable to inhibition than CCL5 interactions due to the fact that CCL20 is a weakly oligomerizing chemokine [159] whereas CCL5 interacts with GAGs as a high affinity polymer with multiple redundant epitopes. In any case, success will be dependent on synthetic feasibility, and, fortunately, there has been enormous progress in carbohydrate chemistry in recent years [43,160,161,162], which may help position GAG mimetics as viable therapeutic entities. 

### 6.3. Antibody-Based Strategies

Although the majority of approaches for therapeutics targeting the chemokine system have been small molecules, several programs have aimed to develop antibodies against chemokines or their receptors. However, the only successful anti-chemokine antibody developed thus far is a topical anti-CXCL8 monoclonal antibody for psoriasis (Abcream, a product of Anogen), which was approved in China. Examples of failures include an anti-CXCL8 antibody produced by Abgenix [163], which did not meet its endpoints in phase II trials for chronic obstructive pulmonary disease and psoriasis, and anti-CCL2 antibodies for rheumatoid arthritis [164] and metastatic prostate cancer [165] which also failed due to lack of efficacy [37]. Many possible reasons for efficacy failures have been proposed. For example, the anti-CXCL8 antibody did not recognize GAG bound CXCL8, which could be hypothesized to be the cause of its failure [163], and the anti-CCL2 caused dose dependent increases of systemic CCL2 [164]. Additionally, the CCL2/CCR2 axis is now considered an inappropriate target for rheumatoid arthritis, which would account for clinical trial failures [166,167]. 

**Figure 6:**
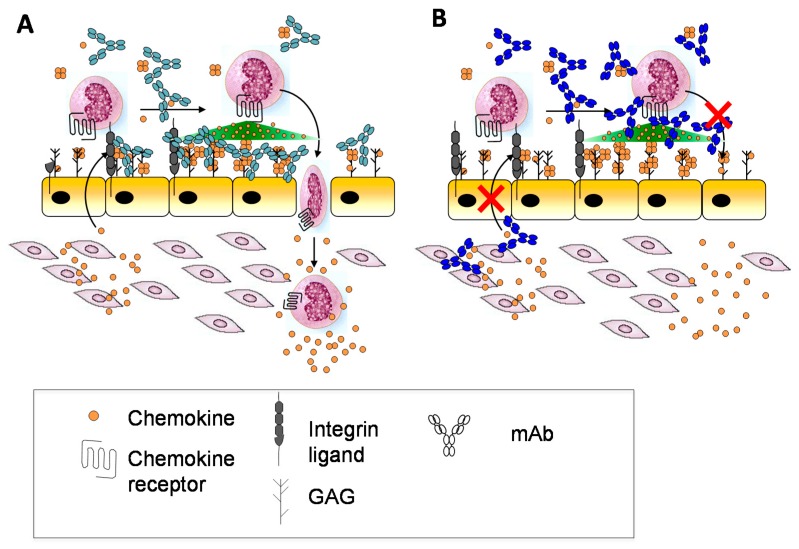
Proposed modes of action of antibodies 1B6 and 1F11. In the updated model of chemokine activity the directional signal is produced by a localized cloud of soluble chemokine. (**A**) The majority of 1B6 molecules are bound to GAG-displayed CXCL10 and free chemokines in the soluble gradient are not inhibited. (**B**) The binding of the antibody to soluble chemokines inhibits cell migration induced by the soluble chemokine cloud and may interfere with the formation of the chemokine gradient.

Other potential reasons may relate to whether the form of a chemokine recognized by an antibody is the appropriate target. As described above, chemokines may be soluble or bound to GAG, and in response to prior anti-chemokine antibody failures, it had been suggested that antibodies targeting both the GAG-bound form and the soluble form of a given chemokine might be most effective [37] (Figure 6A). A comparison of two anti-murine CXCL10 antibodies that recognize soluble CXCL10 (antibody 1B11) versus GAG-bound CXCL10 (antibody 1B6) illustrated how this is indeed an important parameter that needs to be considered, and which form might be most efficacious for blocking the chemokine–receptor interaction [37,168]. In vitro chemotaxis studies showed that 1B6 (recognizing GAG-bound CXCL10) was considerably more potent than 1B11 (recognizing soluble CXCL10). Surprisingly, however, in several in vivo disease models, the less potent 1B11 showed efficacy, whereas 1B6 showed little effect [37]. After ruling out other reasons for the failure of 1B6, it was concluded that complexation of the antibody with GAG-bound chemokine causes target-mediated drug disposition (TMDD) and rapid clearance from the circulation. Furthermore since the amount of the GAG-bound form significantly exceeds that of the soluble form, most of the antibody would be depleted leaving little to neutralize soluble CXCL10. By contrast, binding of 1B11 to soluble CXCL10 inhibits chemokine interactions with both GAGs, required for forming a gradient, and with receptor CXCR3 (Figure 6B) [37]. These data illustrate how the complexities introduced by chemokine–GAG interactions need to be carefully considered when developing antibodies, with special attention to those chemokines that are particularly abundant and avid GAG binders. The data are also most consistent with the “cloud” concept—that CXCL10 must be released from GAGs to bind its receptor on leukocytes in soluble—and they provide further evidence that GAG-bound CXCL10 is not “the active form”.

## 7. Conclusions

Chemokine–receptor interactions are often described as “redundant”, because many chemokines interact with the same receptor and many receptors interact with multiple chemokine [169]. While this may be true to some extent, there are now many examples where GAGs break this otherwise functional redundancy. Although precise in vivo GAG partners for chemokines are not known, it is clear that chemokines have a wide range of affinities for GAGs, ranging from extremely weak binders (e.g., CCL3 and CCL4) to extremely strong nanomolar and subnanomolar binders (CCL5, CCL21, CXCL4, CXCL11). Chemokines also have a wide range of propensities to oligomerize in solution or on GAGs, while others have unstructured C-terminal tails as GAG-binding domains. Similarly, they also have a wide range of propensities to heterodimerize. Without even considering impacts on other functions described in this review, all of these parameters will effect the size, shape and duration of chemokine gradients and whether they are haptotactic or soluble. CCL19 and CCL21 are the most well studied examples of non-redundant ligands of the same receptor, CCR7. Whereas CCL21 has a C-terminal tail that enables it to bind GAGs with high affinity and to form haptotactic gradients, CCL19 lacks the tail and is considered a soluble chemokine. Of the CCR5 ligands, all of which form stable polymers, CCL5 is basic and has high affinity for GAGs whereas CCL3 and CCL4 are acidic and have low affinity for GAGs. CXCL4L1, like CXCL4 is a ligand of CXCR3; however it differs by three amino acids, which is sufficient to make it essentially a soluble chemokine in contrast to the exceptional high affinity of CXCL4 for GAGs [170]. 

From these and other examples, it has become clear that there is a great deal of diversity in chemokine–GAG interactions. However, most of the observations have been made from in vitro studies. Going forward, it will be critical to understand their functional significance in vivo. If this can be done, it may be possible to exploit this information to make better therapeutics, as illustrated by the anti-CXCL10 antibody story where understanding whether one should target soluble versus GAG-bound chemokine was the difference between success and failure in an animal model.
